# Association Between Obesity and Self-Reported Depression Among Female University Students in the United States

**DOI:** 10.7759/cureus.31386

**Published:** 2022-11-11

**Authors:** Oluwasegun A Akinyemi, Olajumoke Babatunde, Terhas Asfiha Weldeslase, Irene Akinyemi, Bolarinwa Akinwumi, Adeolu O Oladunjoye, Temitope Ogundare, Maureen Bezold

**Affiliations:** 1 Health Policy and Management, University of Maryland School of Public Health, College Park, USA; 2 Surgery, Howard University, Washington DC, USA; 3 Pharmacotherapy, Virginia Commonwealth University, Richmond, USA; 4 Surgery, Howard University College of Medicine, Washington DC, USA; 5 Nursing, Spoon River College, Macomb, USA; 6 Health Sciences and Social Work, Western Illinois University, Macomb, USA; 7 Psychiatry, Baylor College of Medicine, Houston, USA; 8 Medical Critical Care, Boston Children's Hospital, Boston, USA; 9 Psychiatry and Behavioral Sciences, Boston University School of Medicine, Boston, USA

**Keywords:** overweight, self-reported depression, college student, association, obesity

## Abstract

Aim: To determine the relationship between obesity and depression among female undergraduate students at Western Illinois University (WIU), Macomb, Illinois.

Methods: A cross-sectional study using self-reported questionnaires were conducted between August 15, 2019, and December 15, 2019. A cohort of 434 female undergraduate students was retrieved from the study. We determined the association between self-reported diagnosis of depression within the last year and body mass index (BMI) among female students.

Results: The prevalence of depression among female undergraduates at WIU was 33.2%. Obese and overweight female undergraduate students had a higher likelihood of being diagnosed with depression than students with normal BMI (reference), overweight (OR= 1.91; 95% CI 1.11-3.31), obese (OR= 2.20; 95% CI 1.30-3.80). Latino and black students were less likely to report depression than white students, Latino (OR=0.37 95% CI 0.15-0.92), and Black (OR= 0.40; 95% CI 0.18-0.86). There was also a positive association between chronic back pain and development of the diagnosis of depression, (OR=2.26; 95% CI 1.45-3.52).

Conclusion: Depression among female undergraduate students is very common in the USA. Obese and overweight female students are more likely to be depressed than students with normal BMI. There is a need for urgent public health interventions to reduce the obesity rate among university students.

## Introduction

Depression is a mental health disorder characterized by persistently depressed mood or loss of interest in activities, causing significant impairment in daily life. It affects women twice as much as men [1] and is a significant cause of reduced quality of life. It was ranked as the world's third most impactful health problem and is projected to be the most impactful chronic disease by 2030 [2]. Its prevalence has recently increased in the United States [3]. Major depression's lifetime prevalence in 2018 was 20.6%, with a 12-month prevalence of 10.4% [4], a significant increase from the lifetime prevalence of 16.6% and the 12-month prevalence of 8.6% reported in 2008 [5].

The prevalence of depression and other mental health disorders has also increased in US colleges in recent years [6]. It is a significant cause of poor sleep quality [7] and self-harm [8] in college students. It is a significant cause of poor academic performance among university students [9], leading to high dropout of school rates [10].

Obesity has continued to increase in the US since the 1970s [11]. According to recent estimates from the Centers for Disease Control and Prevention (CDC), about 93 million adult Americans, which constitutes about 42% of the adult population, are obese [12]. The prevalence of obesity has increased among adolescent females from the 1980s at 10% to 21% in the last few years [13]. The increased prevalence of obesity has also affected college and university students [14]. This is a worrisome trend as obesity is associated with a significant risk of cardiovascular diseases [15], hypertension [16], obstructive sleep apnea [17], and many other chronic diseases. Studies have demonstrated increased mortalities among the obese population [18].

Obesity and depression are major public health problems. The association between obesity and depression is bidirectional [19], with often devastating outcomes. Significant reduction in this association can only be achieved by positive lifestyle changes and improved healthcare delivery [20].

The present study aims to determine the prevalence of depression among Western Illinois University (WIU) female undergraduate students and to find out if there is an association between obesity and depression among this population.

## Materials and methods

Data source and patient selection

We conducted a cross-sectional study with self-reported questionnaires. These questionnaires were generated through Google Forms, and the link was sent to potential participants through the WIU Administrative Information Management System (AIMS). Four hundred thirty-four female undergraduate students participated in the online survey between August and December 2019. 

Main outcome

The primary outcome was self-reported depression in the last year. We defined self-reported depression as a diagnosis by a healthcare worker in the past year before the study. The survey contained a question: "have you been diagnosed by your doctor with depression in the past year?' 

Covariates

The survey also contained questions about students' weight and height, from which we calculated the body mass index (BMI). We stratified the BMI into underweight (BMI < 18.5), normal BMI (BMI: 18.5-24.9), overweight (BMI: 25.0-29.9), and obese (BMI ≥ 30.0). Other information collected was race/ethnicity, stratified as White (Non-Hispanic White), Black (Non-Hispanic Black), Hispanic, and Other. We also classified the students in order of the school year as freshmen, sophomores, juniors, and seniors, with the students' age defined as < 24 years and ≥ 24 years. Other covariates included in our final multivariate analysis were regular physical exercise, school employment, and chronic back pain, which is defined as a complaint of back pain > six months duration.

Ethical approval

The findings were a secondary analysis from an initial study (The Impact of Obesity on the Academic Performance of Western Illinois University Students) approved by the Institutional Review Board of WIU (# 224-19). Only students who signed a written consent form included in the questionnaires participated in the study.

Statistical analysis

We utilized descriptive statistics such as frequencies and percentages to describe students' demographics. Next, we evaluated the relationship between the studied variables and self-reported depression using Pearson chi-square tests. In the final multivariate regression analyses, we estimated the independent association between obesity and the risk of depression diagnosis in the past year. The results were reported as adjusted odds ratios and 95% confidence intervals. A 2-tailed p-value <0.05 was considered statistically significant. All statistical analyses were performed using the SPSS software, version 16.0 (IBM Corp., Armonk, NY).

## Results

Table 1 shows the distribution of socio-demographic characteristics of the study respondents. Among the female undergraduate students who participated in the study, 125 (28.8%) were obese, and 111 (25.6%) were overweight. Only 184 (42.4%) had normal BMI, while 14 (3.2%) students were underweight. 

**Table 1:** Socio-demographics of study participants BMI, Body mass index

Variables	Frequency	Percentages
BMI		
Underweight	14	3.2
Normal	184	42.4
Overweight	111	25.6
Obese	125	28.8
Race/Ethnicity		
Whites	334	77
Blacks	51	11.8
Hispanics	33	7.6
Others	16	3.7
Year in school		
Seniors	142	32.7
Juniors	113	26
Sophomore	48	11.1
Freshmen	131	30.2
Age		
< 24 Yrs.	349	80.4
≥24 Yrs.	85	19.6
Self-Reported Depression		
Yes	144	33.2
No	290	66.8
Regular Physical Exercise		
Yes	339	78.1
No	95	21.9
School Employment		
Yes	224	51.6
No	210	48.4
Chronic Back pain		
Yes	189	43.5
No	245	56.5

Most respondents were White, 334 (77%), Black students accounting for 11.8%, and Hispanic students, 33 (7.6%). Based on the year in school, 142 (32.7%) of the study respondents were in their Senior years, 113 (26.0%) were Juniors, 48 (11.1%) were Sophomores, and the remaining 131 (30.2%) were Freshmen. 

The study respondents were stratified into two groups based on age. There were 349 (80.4%) students who were less than 24 years, with the remaining 85 (19.6%) at least 24 years or above. There were 144 (33.2%) students who reported a clinical diagnosis of depression in the last year preceding the survey, while the remaining 290 (66.8%) had no such history. The majority of the study respondents, 224 (51.6% had on-campus employment, with the reaming 210 (48.4%) not having on-campus employment. A significant proportion of the study respondents 189 (43.5%) reported a history of chronic back pain in the last year preceding the survey, and the remaining 245 (56.5%) had no such history. 

Table 2 shows an association between the occurrence of depression in the last 12 months before the study and socio-demographic variables. There was a significant association between increasing BMI and the occurrence of depression. The obese and overweight group had the most significant proportion of students with a clinical diagnosis of depression in the last year preceding the study, p < 0.05. 

**Table 2:** Association between self-reported depression and socio-demographic variables * p < 0.05, statistical significance BMI, Body mass index, CGPA, cumulative grade point average.

Variables	Depression	No Depression	X2	df	p-value
CGPA	3.75 ± 0.16	3.73 ± 0.17			0.16
BMI			14.97	3	<0.001*
Underweight	5 (35.7%)	9 (64.3%)			
Normal	44 (23.9%)	140 (76.1%)			
Overweight	39 (35.1%)	72 (64.9%)			
Obese	56 (44.8%)	69 (55.2%)			
Chronic Back pain			14.14	1	<0.001*
Yes	81 (42.9%)	108 (57.1%)			
No	63 (25.7%)	182 (74.3%)			
Year in school			6.2	3	<0.001*
Senior	57 (40.1%)	85 (59.9%)			
Junior	37 (32.7%)	76 (67.3%)			
Sophomore	16 (33.3%)	32 (66.7%)			
Freshmen	34 (26.0%)	97 (74.0%)			
Race/Ethnicity			10.58	4	0.03*
Whites	124 (37.1%)	210 (62.9%)			
Blacks	10 (19.6%)	41 (80.4%)			
Hispanics	7 (21.2%)	26 (78.8%)			
Others	3 (21.4%)	11 (78.6%)			
Age			4.01	1	0.03*
< 24 yrs.	108 (30.9%)	241 (69.1%)			
≥ 24 yrs.	36 (42.4%)	49 (57.6%)			
School employment			0.91	1	0.2
Yes	79 (35.3%)	145 (64.7%)			
No	65 (31.0%)	145 (69.0%)			
Regular Physical exercise			0.74	1	0.39
Yes	109 (32.2%)	230 (67.8%)			
No	35 (36.8%)	60 (63.2%)			

Students with a history of back pain were more likely to be depressed, as shown in the table. 42.9% of students with complaints of back pain were depressed compared to only 25.7% of students without a history of back pain, p < 0.05.

White students had the largest proportion of depression compared to other races; 37.1% of white students were depressed compared to 19.6% of Blacks and 21.2% of Hispanics. This difference in the prevalence of depression among the different races was statistically significant, p < 0.05. There was a statistically significant association between the age of respondents and the occurrence of depression, with students who were at least 24 years more likely to be depressed than those younger than 24 years, p < 0.05. There was no significant association between on-campus employment, physical exercise, or CGPA, and self-reported depression, p > 0.05. 

Table 3 is a multivariate analysis of possible predictors of depression among study respondents. Female undergraduates who are obese have a two-fold increased likelihood of reporting depression in the last year preceding the survey compared to those with a normal BMI. Also, overweight female students have a 1.9-fold likelihood of reporting depression compared to their counterparts with normal BMI. Female undergraduates who are Latino or black have a 60% lower likelihood of reporting depression in the last year compared to their white counterparts. 

**Table 3:** Predictors of self-reported depression among female college students * p < 0.05, level of significance BMI, Body mass index, CGPA, cumulative grade point average.

Variables	Odds ratio	95% Confidence Interval		p-value
		Lower CI	Upper CI	
Age category				
≥ 24	Reference			
< 24	0.964	0.552	1.682	0.91
BMI				
Normal BMI	Reference			
Underweight	1.906	0.575	6.316	0.29
Overweight	1.92	1.107	3.332	0.02*
Obese	2.225	1.298	3.813	0.04*
Race/ethnicity				
Whites	Reference			
Blacks	0.396	0.183	0.859	0.02*
Hispanics	0.366	0.146	0.923	0.03*
Others	0.443	0.113	1.734	0.24
School year				
Freshman	Reference			
Junior	1.341	0.711	2.528	0.36
Sophomore	1.598	0.727	3.511	0.24
Senior	1.698	0.914	3.156	0.09
CGPA	2.481	0.637	9.668	0.19
Chronic Back Pain	2.257	1.445	3.524	<0.001*
School Employment	0.993	0.626	1.575	0.98
Regular Physical Exercise	0.922	0.545	1.56	0.76

Finally, female students with a history of chronic back pain have a two-fold increased likelihood of depression than those students who did not have this complaint. The association between age and depression became insignificant when we adjusted for other factors.

## Discussion

The study demonstrated an association between obesity and self-reported depression among female undergraduates attending WIU. In addition, we reported a strong association between students’ race/ethnicity and premorbid chronic back pain and incidence of depression. Black and Latino students were less likely to have a diagnosis of depression in the year preceding the study than their white counterparts. Students who reported having a diagnosis of depression in the past year had a higher BMI than those who did not report a depression diagnosis. Previous studies have reported similar findings [21,22]. 

The relationship between obesity and depression is bi-directional [23,24]. Obesity is associated with stigma, predisposing to depression, especially among female students [25]. In addition, physical limitations caused by obesity or associated comorbidities can increase the risk of depression [20]. Also, depression is associated with increased appetite, binge eating, and a reduction in exercise, all of which can contribute to obesity [26]. In addition, certain medications used to treat depression can increase the risk of obesity [27]. 

The present study did not find any association between regular physical activity and depression. Several studies have demonstrated that those who exercise regularly are less likely to have depression [28,29]. The present findings are in line with a study by Hawker et al. They reported that among student nurses at Cardiff University, there was no association between physical activity and depression [30]. Our inability to detect an association between physical activity and depression may also be due to the methodology of the present study. Studies that saw an association between physical activity and depression often used extensive samples and standardized questionnaires to measure physical activity.

Similar to the findings in this study, studies have demonstrated that Blacks and Hispanics are less likely to report depression [31,32] compared to their white counterparts. However, this finding is counterintuitive as minority groups are exposed to more social stressors [33,34]. A significant explanation may be the belief that minority groups are often more religious, therefore, they are more hopeful and receive more social support than whites [35]. 

Finally, we reported an association between preexisting chronic back pain and an increased risk of depression. This finding is similar to other reports in the literature [36]. The presence of chronic illnesses increases the risk of depression, usually from the stigma, the limitation in activity, loss of productivity and reduction in quality of life, the need for constant medications, and the associated side effects of drugs, all of which contribute to increasing the risk for developing depression [37]. 

Some limitations of this study include the use of self-reported diagnosis of depression, which may have introduced recall bias or social attribution errors that may affect the true prevalence of depression among this population. In addition, its cross-sectional nature could not demonstrate a causal relationship between obesity and depression. Future studies should include a larger sample size and use standardized instruments to measure depression. 

## Conclusions

In the present study, we have demonstrated a high prevalence of self-reported depression among overweight and obese female college students. The higher prevalence of self-reported depression among these students may influence their overall quality of life. Therefore, there is a need for a lower threshold for the diagnosis of depression in college students and also innovations in interventions to improve mental health and wellness. Given the link between obesity and depression, targeted interventions to promote weight loss and a healthy lifestyle among college students will be beneficial in reducing the risk of depression among this population.
